# A Unique Case: An Immense Fibroid, Involving Uterus and Ovaries, with Extensive Adhesions; Exploratory Laparotomy

**Published:** 1887-04

**Authors:** A. W. Acheson

**Affiliations:** Denison, Texas


### A UNIQUE CASE.

### AN IMMENSE FIBROID, INVOLVING UTERUS AND OVARIES, WITH EXTENSIVE ADHESIONS; EXPLORATORY LAPAROTOMY.

*By A. W. Acheson, M. D., Denison, Texas.*

For Daniel's Texas Medical Journal.

THE case of Mrs. Emma Carse is interesting from the fact that a previous operation had been attempted, but abandoned for reasons which could not be learned. After the first operation, she got out of bed as soon as her physicians left the room, in doing which she tore out one or two of the stitches. The wound remained gaping for some ten hours before her bandages were unloosed to repair it. In remonstrating with the surgeons for not completing the operation, she learned that the attachments of the tumor were too great to justify them in proceeding; but had they known she could endure so much exposure of the peritoneal cavity as she had undergone, they would have proceeded.

She sought surgeon after surgeon during the next few years, even going to Philadelphia to consult Dr. Gross, for the purpose of having the tumor removed. But all declined to interfere, partially because of previous failure, but chiefly because she allowed the surgeon no latitude of judgement. Her idea was, to remove that tumor, even if she died upon the table. Her life was of secondary importance, and must be so considered by the surgeon. Failing, after years of search, to find anybody willing to operate under these conditions, she concluded to submit to an exploratory operation, with the assurance that if it were possible the tumor should be removed, but if not, the operation to be abandoned.

February 12, 1882, an incision was accordingly made in the linea alba about fourteen inches in length, so as to satisfactorily expose the abdominal contents. Adhesions were found so numerous, particularly around the bladder, that the operation was discontinued.

Four hours after transferring her from the table to the bed she was visited, and found sitting up. When re-told of the danger of throwing all the weight of the abdominal contents on the stitches, she stated that she got out bed an half hour after the doctors left, and waved her handkerchief from the window to some friends on the train. When again cautioned with regard to the danger of getting up, she promised to lie down, and stay there; yet I learned afterwards, that she sat up more than half her time; that she planted her garden in potatoes at the end of the first week, and that she climbed upon the back fence, seven feet high, on the eighth day.

On October 28, 1884, she died. Almost her last expression was: "Thank God I don't have to be buried with this bunch. Doctor, recollect your promise."

A post-mortem was held next day and a fibrous tumor, including both womb and ovaries, was removed, weighing over forty pounds.

Dr. John W. Jackson operated, assisted by Dr. Carson, of St. Louis, and myself.

This case has not been before reported.

---

Induce every M. D. to subscribe for DANIEL'S TEXAS MEDICAL JOURNAL. Only $2 a year, in advance.

